# On the Recent Use of Membrane Technology for Olive Mill Wastewater Purification

**DOI:** 10.3390/membranes5040513

**Published:** 2015-09-28

**Authors:** Javier Miguel Ochando-Pulido, Antonio Martinez-Ferez

**Affiliations:** Department of Chemical Engineering, University of Granada, Granada 18071, Spain; E-Mail: amferez@ugr.es

**Keywords:** olive mill wastewater, membrane processes, microfiltration, nanofiltration, reverse osmosis, membrane bioreactors

## Abstract

Many reclamation treatments as well as integrated processes for the purification of olive mill wastewaters (OMW) have already been proposed and developed but not led to completely satisfactory results, principally due to complexity or cost-ineffectiveness. The olive oil industry in its current status, composed of little and dispersed factories, cannot stand such high costs. Moreover, these treatments are not able to abate the high concentration of dissolved inorganic matter present in these highly polluted effluents. In the present work, a review on the actual state of the art concerning the treatment and disposal of OMW by membranes is addressed, comprising microfiltration (MF), ultrafiltration (UF), nanofiltration (NF), and reverse osmosis (RO), as well as membrane bioreactors (MBR) and non-conventional membrane processes such as vacuum distillation (VD), osmotic distillation (OD) and forward osmosis (FO). Membrane processes are becoming extensively used to replace many conventional processes in the purification of water and groundwater as well as in the reclamation of wastewater streams of very diverse sources, such as those generated by agro-industrial activities. Moreover, a brief insight into inhibition and control of fouling by properly-tailored pretreatment processes upstream the membrane operation and the use of the critical and threshold flux theories is provided.

## 1. Introduction

**Figure 1:**
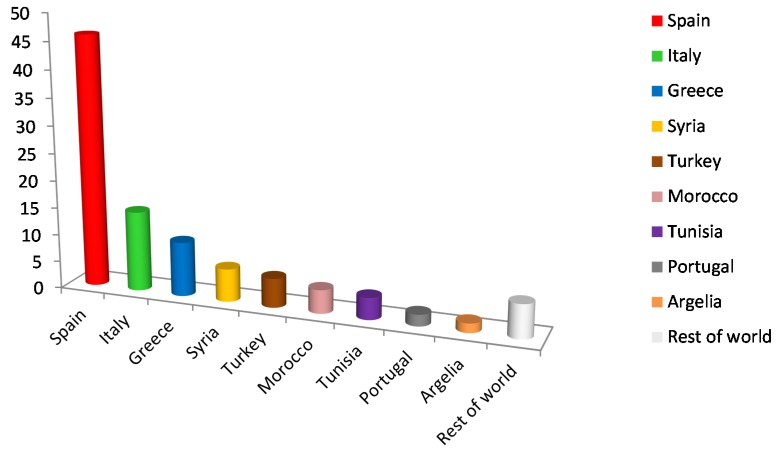
Olive oil production worldwide (data from International Olive Oil Council, IOOC 2014) [1].

The olive oil sector has represented since several decades one of the most important industries in the Countries of the Mediterranean River Basin. Spain, Italy, Portugal, Greece and the Northern African countries—Syria, Algeria, Turkey, Morocco, Tunisia, Libya, Lebanon—cope with the major production worldwide (Figure 1). Other countries such as France, Serbia and Montenegro, Macedonia, Cyprus, Egypt, Israel, and Jordan also present a considerable annual olive oil yield, according to data from the International Olive Oil Council (IOOC, 2013−2014) [1]. Moreover, olive oil production is also becoming an emergent agro-food industry in China and other countries such as the USA, Australia, and the Middle East. It is very worth highlighting the case of China, which exhibits favorable edaphoclimatic conditions for the growth of olive trees, and is expected to develop a significant olive oil production potential in the near future. Hence, the treatment of the olive mill effluents is already a task of global concern and it is no longer a problem limited to a specific region.

Indeed, in recent decades a deep growth of the olive oil industrial sector has been experienced as a result of the modernization of olive oil mills, in response to the increasing demand of olive oil worldwide. Taking the case of Spain, there are more than 1,700 olive mills currently authorized and operating [1]. The production of olive oil employs a very significant number of people and is one of the main industrial activities, as in other countries of the Mediterranean Basin and Southern Europe. More than 1,400,000 tons of olive oil were produced in Spain during the 2013–2014 campaign, 70% of which were obtained in Andalucía where there are 850 olive mills, which yielded a production of 1,022,000 tons of olive oil as well as 4,778,451 tons of table olives, highlighting the provinces of Jaén, with more than 515,000 tons of olive oil and 2,229,000 tons of olives; Granada with 118,000 tons of olive oil and 509,383 tons of olives; and Córdoba with 219,000 tons of olive oil and 1,123,800 tons of olives [1].

The significant boost of this industrial sector in the last years has brought an undesired side-effect: the amounts of olive mill wastewaters (OMW) by-produced have also significantly increased, especially as a result of the change of the antique batch press method for the continuous centrifugation-based olive oil production processes currently used, which ensure much higher productivity (Table 1 and Table 2). On one hand, these continuous systems guarantee a higher yield in recovering olive oil from the olives, up to 21%, but on the other they lead to an increased production of wastewater streams due to water injection in the centrifugation processes [2]. An average-sized modern olive oil factory currently generates between 10 and 15 m^3^ daily of OMW derived from the proper extraction process, and also 1 m^3^ of wastewater derived from the washing of the olives per processed ton (olive washing wastewater, OWW) [2]. This raises several million cubic meters of these effluents annually, which represents a huge amount of these highly contaminant wastewaters as well as of potable water consumption.

**Table 1:** Characteristics of the effluents of batch and continuous olive oil extraction processes [3].

Process	Effluent	COD, g/L	BOD_5_, g/L	TSS, g/L	pH	EC, mS/cm	TPh, g/L
Olive cleaning	OWW	0.8–2.2	0.3–1.5	8–18	5.5–6.6	2.5–3.0	0–0.1
Batch press	OMW-P	130–130	90–100	10–12	4.5–5.0	2.0–5.0	1.0–2.4
Three phase	OMW-3	30–220	5–45	5–35	3.5–5.5	2.0–7.9	0.3–7.5
Two phase	OMW-2	4–18	0.8–6.0	2–7	3.5–6.0	1.5–2.5	0.1–1.0

COD: chemical oxygen demand; BOD_5_: biochemical oxygen demand; TSS: total suspended solids; EC: electric conductivity; TPh: total phenolic compounds.

**Table 2:** Effluents flowrates of continuous olive oil extraction processes [2].

Effluent, L/kg	3-phase extraction	2-phase extraction
Washing of olives (OWW)	0.06	0.05
Horizontal centrifuge	0.90	0
Vertical centrifuge	0.20	0.15
Cleaning	0.05	0.05
*Total*	1.21	0.25

The current necessity of maximizing the production processes too often excludes the planning of the environmental protection. Wastewater treatment for ulterior uses in multiple applications contributes to sustainable water consumption and the regeneration of the public hydraulic domain and the ecosystems. In this scenario, the European Directive 2000/60/CE took the lead in establishing the legal framework to confer the utmost protection to water, highlighting the use of regenerated wastewater. Moreover, European Environmental Regulations will become more stringent in virtue of the ‘H2020 Horizon’ (http://ec.europa.eu/programmes/horizon2020/). 

Direct discharge of OMW has been reported to cause strong odor nuisance, soil contamination, inhibition of plant growth, hindrance of self-purification processes, underground leaks, and water body pollution, as well as severe impacts to the aquatic fauna and to the ecological status [3,4,5]. Discharge of untreated OMW to the ground fields and superficial water bodies is actually prohibited in Spain, whereas in Italy and other European countries only partial discharge on suitable terrains is allowed. Because of the presence of high levels of organic pollutants and refractory compounds, direct disposal of these effluents to the municipal sewage treatment systems is also prohibited. Legal limits are established in order to prevent difficulties to the municipal sewer treatment plants, which operate on the basis of biomasses that must be maintained alive.

OMW exhibit a series of characteristics that make their reclamation by conventional physicochemical treatments utterly difficult. The presence of phytotoxic recalcitrant pollutants—such as phenolic compounds, organic and long chain fatty acids, tannins and organohalogenated contaminants—makes these effluents resistant to biological degradation and thus inhibits the efficiency of biological processes. Furthermore, the physicochemical composition of OMW is extremely variable as it depends on several factors such as the extraction process, edaphoclimatic and cultivation parameters, as well as the type, quality and maturity of the processed olives, among others. OMW typically exhibit intense violet-dark colour, acid pH, strong odor, high saline toxicity reflected in the electroconductivity values, and very heavy organic pollutants load.

A focus on Table 1 shows that the pollutants concentration levels in the effluent from the washing of the olives (OWW) stands normally below the standard limits for discharge on superficial suitable terrains (e.g., data from the Guadalquivir Hydrographical Confederation, 2006–2014: TSS < 500 mg/L and COD < 1000 mg/L) (Table 3) [6]. However, concentration values might exceed the established standards, and this fact depends on the water flowrate used in the olive washing machines during the fruit cleaning procedure. 

**Table 3:** Parametric standard limits for discharge of OMW in suitable terrains (data from the Guadalquivir Hydrographical Confederation, 2006–2014) [6].

Parameter	2002	2003	2004	2006	2014
pH	6–9	6–9	6–9	6–9	6–9
TSS, mg/L	600	500	500	500	500
COD, mg/L	2,500	2,000	1,500	1,000	1,000
BOD_5_, mg/L	2,000	-	-	-	-

On the other hand, major organic pollutants load is commonly measured in the effluent coming out of the centrifuges (OMW-2 and OMW-3), and most of them are phytotoxic compounds recalcitrant to biological degradation. Therefore, the presence of these substances would be hardly reflected by biochemical oxygen demand (BOD_5_) measurements, thus the chemical oxygen demand (COD) seems a more appropriate parameter together with total phenolic compounds (TPh) concentration, as reflected in Table 3.

Taking into consideration that in the two-phase extraction process water injection is only practiced in the final vertical centrifugation step, the volume of liquid effluent derived from the production process (OMW-2) is reduced by one-third on average with respect to the amount required for the three-phase system. Moreover, much of the organic matter remains in the solid waste, which contains higher moisture than the pomace from the three-phase system (60–70% in two-phase systems vs. 30–45% in three-phase ones, OMW-3) and hence OMW-2 exhibits a lower degree of pollutants, too: the measured chemical oxygen demand (COD) in OMW-2 is commonly in the range 4–18 g/L in contrast with up to 30–220 g/L in case of OMW-3. Inorganic compounds including chloride, sulfate, and phosphoric salts of potassium, calcium, iron, magnesium, sodium, copper, and traces of other elements are other common traits of OMW [3,4,5].

The disposal of the solid waste is not within the scope of the present work, which is focused only towards the management of the problem related to the reclamation of the liquid effluents. Some examples of solutions already proposed for the management of the pomace waste are adsorption of heavy metals [7,8], dyes [9], and phenols [10], as well as composting [11] or biogas production [12], among others.

The small size and geographical dispersion of olive oil mills as well as the seasonality of olive oil production represent the main obstacles for the implementation of cost-effective management processes for these effluents. A centralized treatment of OMW does not seem feasible currently, thus an effective and simple solution is needed for these small plants. 

Many reclamation treatments and integrated processes for OMW have already been proposed and developed but not led to completely satisfactory results, such as lagooning or natural evaporation and thermal concentration [13], composting [14], treatments with clay or with lime [15,16], physico-chemical procedures such as coagulation-flocculation [17,18], and biosorption [19], advanced oxidation processes including ozonation [20], Fenton´s reaction [21,22] and photocatalysis [23,24], electrochemical treatments [25,26], and hybrid processes [27,28,29,30].

Furthermore, because of the significant salinity of OMW, reflected in their high electroconductivity (EC) values, conventional physicochemical treatments are not effective. These treatments are not able to abate the high concentration of dissolved ionic concentration present in these effluents, which present hazardous salinity levels according to the guidelines established by the Food and Agricultural Organization (F.A.O.) for irrigation purposes (Table 4).

In order to attempt the complete depuration of OMW, membrane technology offers compact modular nature, high efficiency, and moderate investment and maintenance expenses. Membrane processes are becoming extensively used to replace many conventional processes in the purification of water and groundwater as well as in the reclamation of wastewater streams of very diverse sources, such as those generated by agro-industrial activities [31,32,33,34,35,36,37]. The availability of new membrane materials, designs, module concepts and know-how has promoted credibility among investors.

In the present work, a review on the actual state of the art of the treatment and disposal of the OMW from both the two-phase and three-phase systems by membranes will be addressed, comprising microfiltration (MF), ultrafiltration (UF), nanofiltration (NF), and reverse osmosis (RO), as well as membrane bioreactors (MBR) and other non-conventional membrane processes.

**Table 4:** Irrigation water quality as a function of its salinity (Food and Agriculture Organization, FAO).

EC, dS/cm	Water quality	Risk due to salinity
0–1	Excellent to good	Low to mid
1–3	Good to marginal	High
> 3	Marginal to unacceptable	Very high

## 2. Membrane Treatment of Three-Phase Olive Mill Wastewater

The biggest technical drawback for the implementation of membrane technologies in wastewater treatment plants is the high fouling potential. Membrane fouling—which can be caused by colloids, soluble organic compounds, and microorganisms that are typically not well removed with conventional pretreatment methods—increases the feed pressure and requires frequent membrane cleaning procedures. Concerning this, properly-tailored pretreatment processes are required in order to avoid high fouling rates, which would rapidly lead to zero flux conditions if no pretreatment is conducted on the raw effluent upstream the membrane operation. 

Turano *et al.* [38] proposed an integrated centrifugation-UF process for the treatment of OMW from three-phase centrifugation-based olive mills in Italy. The preliminary centrifugation step achieved the removal of suspended solids, and the centrifuge supernatant was conducted to UF (polysulfone, MWCO 17 kDa). It was reported up to 55% COD reduction was achieved after the proposed centrifugation pretreatment, whereas 80% removal of suspended solids concentration and 90% COD reduction was achieved at the outlet of the integrated process. Moreover, centrifugation is a simple and mechanical operation, which does not need addition of chemicals, and it is available in the production line.

Paraskeva *et al.* [39] investigated the treatment and complete fractionation of OMW coming from a three-phase-decanter olive oil mill in Greece, using a combination of different membrane processes. The authors tested UF followed by NF and/or RO in a batch mode, keeping constant the composition of the initial raw wastewater.

Prior to UF, screening with an 80 μm polypropylene filter was performed to remove suspended solids. The UF membrane was made of ceramic material (zirconia, mean pore size 100 nm) in multichannel configuration. The recovery ratio was fixed between 80–90% of the initial OMW volume, at 15–35 °C operating temperature and TMP between 1.0 and 2.25 bar. UF succeeded in the separation of high molecular weight constituents including suspended solids, as well as the condensation of solid lipid components (up to 90%) and a large amount of the phenolic compounds (~50%).

Polymeric membranes in spiral wound configuration were used for either NF (200 Da MWCO) or RO (100 Da MWCO) tests, in order to further treat the UF permeate. In NF tests, the temperature was kept constant at 20 °C, and the best TMP (20 bar) led to satisfactory permeate flowrate between 100–120 L/h. Following the NF step, phenols were removed to an extent exceeding 95% of the initial value, but even better efficiency was achieved by applying RO after NF, which enabled a significant conductivity, salinity, and turbidity decrease and nearly 30 L/h permeate flowrate. Best performances were found to occur at 35 °C and high-pressure values (TMP = 40 bar), which allowed reaching a turbidity value of 14 NTU and a decrease of up to 98.9% of the raw water conductivity, with a recovery between 75–80% of the initial OMW volume.

Finally, they stated not only that the chemical composition of the post-treated effluent was suitable for disposal in aquatic receptors or for irrigation purposes, but also that both the inorganic part of OMW (N, P, Mg, K, metal traces) and the organic fraction (hydrocarbons, nitrogenous compounds, organic acids, polyalcohols) may be used as plant nutrients, perhaps in combination with other inorganic or organic fertilizers such as manure or sludge from biological treatments of other types of wastes [40,41]. On the other hand, polyphenols and fats ought to be separated, as described before, due to their phytotoxic properties [42,43]. Nonetheless, no data on the permeate flux decline regarding the operating conditions was reported and fouling was not discussed by the authors.

Stoller and Chianese [44,45,46] studied the purification of the wastewater stream generated in the olive washing procedure (OWW) to comply with discharge standards in municipal sewers (Italy). In contrast with OMW, this effluent presents a moderate organic pollutants load, but high concentration of suspended solids. Therefore, the authors proposed a treatment process comprising an initial solid/liquid (S/L) separation stage, followed by batch UF and NF in series (composite thin-film spiral-wound membranes). As S/L separation operation they addressed the use of coagulation-flocculation with polyelectrolytes: aluminum sulfate (AS) or aluminum hydroxide (AH). The two pretreatment processes performed similarly with regard to COD and BOD_5_ rejection efficiencies, but higher productivity was observed in the subsequent membranes-in-series process after flocculation with AS.

In a following research work, Stoller and Bravi [34] applied the same coagulant-flocculants to pretreat three-phase OMW before batch MF, UF, and NF membranes in sequence, and a final RO step. In addition, they examined two other pretreatment processes: photocatalysis (PC) with nanometric titanium dioxide anatase powders irradiated by UV light, and aerobic digestion (AD). To sum up, all pretreatment processes successfully tested down to RO provided final permeate streams with COD equal to 456 mg/L, 242 mg/L, and 385 mg/L (AS, BIO+AS and PC, respectively), complying with irrigation quality standards. However, UV/TiO_2_ photocatalysis performed more efficiently, showing the highest membrane productivity within the shortest residence time (24 h). Coagulation-flocculation residence time was 72 h for both coagulant-flocculants tested. A significant reduction of polyphenols and final dry matter was attained with both coagulants, but with AH the process had to be stopped at the UF step since cost-effective permeate flow rates were not observed due to quick fouling build-up on the membrane. Otherwise, much longer residence time (seven days) was needed for biodigestion. 

Following this, Stoller [47] conducted a deeper study on how flocculation as pretreatment affected the performance of MF, UF, NF, and RO membranes in the treatment of three-phase OMW, by examining the particle size distribution in the effluent of each stage. Stoller underlined the effect created by a secondary flocculation induced by the AS flocculant-derived salts accumulating near the membrane surface, which gathered particles together into bigger aggregates that could be more easily carried away by the tangential flow, thus sensibly reducing fouling.

In sum, in these studies Stoller underlined the key importance of the pretreatment and highlighted that higher pollutants reductions, e.g., in the COD value, do not guarantee the utmost suitability of the adopted pretreatment process. The fact that the pretreatment brings off pollutant particle size (d_p_) far away from that of the membrane pores (D_p_) is of paramount importance to further enhance the steady-state flux values. In conclusion, a non-linear relationship between the critical flux value and the pore-blocking particles density was remarked.

In another study, Akdemir and Ozer [48] proposed a membrane treatment process based on UF (MWCO 30–100 kDa) preceded by pH adjustment (acidic or alkaline) and cartridge filtration (20 μm). In detail, the pretreatment reached 63% COD removal. These authors reported that the highest permeate flux productivity obtained with the 100k Da UF membrane could be attained upon operating conditions of 200 L/h flow rate and an operating pressure equal to 4 bar. However, an operating pressure of 1 bar was observed to be more adequate in order to preserve the UF membrane from fouling issues.

Finally, the COD, TOC, SS, and oil and grease concentrations in the stream exiting the proposed treatment procedure were equal to 6.4 g/L, 2.5 g/L, 320 mg/L and 270 mg/L, respectively, corresponding to removal ratios of 92.3%, 92.7%, 97.1%, and 98.9%. However, the pursued compliance with Turkish standards for wastewater discharge into sewers was not achieved.

Coskun *et al.* [49] investigated the treatment of three-phase OMW (Turkey) previously centrifuged, then filtered via UF membranes followed by NF, and finally RO membranes. The fluxes reached values of up to 21.2–28.3 L/m^2^h for the NF membranes whereas 12.6–15.5 L/m^2^h for the RO membranes, respectively. The maximum COD removal efficiencies obtained at 10 bar ranged from 59.4–79.2% for the NF membranes, whereas between 96.2% and 96.3% for the RO membranes, respectively. For these latter RO membranes, the conductivity removal efficiencies obtained at 25 bar ranged between 93.2% and 94.8%, respectively. One aspect not considered by these authors was the effect of the fouling issues on the steady-state and long-run performance of the used membranes, which was not reported but should be further investigated.

Zirehpour *et al.* [50] examined an integrated MF-UF–NF membrane system for the purification of three-phase OMW in Iran. The effluent was pre-filtered by three-step MF in series, with nominal pore sizes of 50, 5 and 0.2 µm, in concentration mode, subsequently followed by two and three UF and NF membranes, respectively. 

One positive aspect of this study is that the authors did perform an analysis of the fouling behavior of the used UF and NF membranes, by assessment of the flux recovery ratio and degree of the total flux loss during volume reduction factor experiments. The commercial UF membrane provided higher permeate flux than the lab-made one, but it was pointed that the antifouling properties and rejection efficiency of the latter were significantly better. A specific arrangement of the integrated membrane system was concluded to be the UF membrane followed by two-step NF membranes in series, the first NF step providing high flux while the second one providing high rejection.

Di Lecce *et al.* [51] recently investigated the fractionation of OMW (three-phase, Italy) using a two-step MF (tubular polypropylene) and NF (polyamide thin-film composite spiral wound) membrane process at pilot scale. Previously, the OMW samples were filtered through a cotton fabric filter to reduce the suspended solid concentration from 5.4% to 3.8% (w/w). Results revealed a rejection of the NF membrane towards COD, dry matter, phenolic compounds and antioxidant activity higher than 98%, independently of the volume concentration factor. Finally, the purified NF permeate stream obtained presented COD and phenolic contents values very close to those requested for discharge into surface waters. Nevertheless, no dynamic behavior of the membrane system was reported by the authors, nor the permeate flux profile vs. the operation time, nor the fouling build-up rates on the used membranes, which are crucial prints in order to balance the economic viability of the proposed treatment process.

## 3. Membrane Treatment of Two-Phase Olive Mill Wastewater

It is worth pointing out that the existing studies report mainly membrane treatment processes for OMW exiting olive mills operating with the three-phase olive oil production technology, but few membrane studies on OMW generated by olive factories working with the two-phase extraction technology can be found. Also, the existing research works are focused principally on the use of UF and NF membranes, yet there is a gap of knowledge on RO purification of OMW.

Ochando-Pulido *et al.* [35,36,37] studied the reclamation of two-phase OMW (Spain) by RO on a bench-scale, to achieve the quality to recirculate the final effluent to the olives washing machines of the manufacture process to finally close the loop. The raw OMW was previously subjected to an advanced oxidation process (AOP) based on homogeneous Fenton-like reaction [21,22]. This AOP achieved high abatement of pesticides as well as polyphenols and tannins, besides other organic non-humic and humic contaminants associated to OMW. These compounds are resistant to biological degradation and phytotoxic, and have also demonstrated development of fouling on the membranes. The authors highlighted high and stable permeate flux was provided upon recirculation of a fraction of the permeate stream. Under those conditions, 100% suspended solids, phenols and iron removal was achieved, in addition to 99.4% and 98.2% overall COD and conductivity rejection efficiencies, respectively.

On the other hand, Ochando-Pulido *et al.* [3] studied a batch membranes-in-series processes, in detail UF followed by NF and RO, for the reclamation of two-phase olive mill wastewater (OMW-2). In this work, a pH-T flocculation process followed by photocatalysis with ferromagnetic-core titanium dioxide under ultraviolet irradiation (UV/TiO_2_) was examined as pretreatment. The adoption of this treatment sequence helped in reducing the required membrane area, equal to 104.6 m^2^ and 81.4 m^2^ for the UF and NF membranes, respectively, leading to a limited need of overdesign of the membrane plant. In addition, the use of the applied UV/TiO_2_ photocatalysis process enhanced the productivity and longevity of both membranes, and permitted achieving a final treated effluent stream compatible for irrigation purposes.

Furthermore, Ochando-Pulido *et al.* [52] proposed another integrated batch UF and NF membranes-in-series process for the simultaneous treatment of the two main effluents generated in olive mills operating with the two-phase technology, in particular wastewater derived from the washing of the olives (OWW) and from the olive oil washing during the vertical centrifugation (OMW). Beforehand, the raw effluent, that is, 1:1 v/v mixture of OWW and OMW, designed as OWMW-2, was pretreated by pH-temperature flocculation followed by photocatalysis with TiO_2_ nanoparticles under UV irradiation. Significant and stable fluxes were observed on both UF and NF membranes, 15.5 and 22.2 L/m^2^h, respectively. Finally, the treatment line just comprising UF preceded by pH-T flocculation and UV/TiO_2_ photocatalysis provided an effluent compatible for irrigation and permitted the minimum membrane plant dimension, with only six total modules.

## 4. The Use of Membrane Bioreactors for OMW Reclamation

The main handicaps in the treatment of OMW by membrane bioreactors (MBRs) are basically, on one hand, the high residence times required, and on the other hand, the high load of recalcitrant organic compounds present in this type of effluent, that typically hinder the efficiency of biological processes; moreover, the high organic matter concentration in OMW also multiplies the fouling propensity of MBR systems, principally because it represents a good culture medium for deleterious bio-fouling, which appears in the form of gel layers (mainly caused by extracellular polymeric substances) and pore blocking caused by microorganisms as well as the organic particles themselves.

The feasibility of the use of MBRs for the treatment of OMW (three-phase, Tunisia) was examined in a research study by Dhaouadi and Marrot [53]. These authors used an external ceramic UF MBR with 0.1 μm MWCO. The experimental study was carried out with various diluted OMW solutions continuously fed to the reactor, in order to permit the employed biomass to become gradually acclimated. It is worth highlighting that, by using backpulse combinations (1 s/1 min), a significant and stable permeate flux (100 L/hm^2^) could be successfully attained, with zero suspended solids and almost no phenolic compounds. Moreover, backpulsing enabled maintaining a constant permeate flux over a period of several days.

In another study, Conidi *et al.* [54] proposed an integrated membrane treatment for three-phase OMW in Italy, comprising MF (0.2 μm) followed by UF (10 kDa MWCO) membranes and a final MBR, to achieve the reclamation and valorization of OMW from a three-phase olive oil production process in Italy. The MF membrane permitted the removal of suspended solids, after which low molecular weight polyphenolic compounds could be recovered in the UF permeate stream.

The novelty of this study resides in the fact that the UF permeate was finally driven to a multiphase biocatalytic MBR, where the isomer of oleuropein-aglycon was isolated from the phenolic fraction produced during the former oleuropein hydrolysis reaction step, achieving a maximum conversion of 45.7%. This may give a sensible boost to the treatment of OMW, counterbalancing the energy costs.

## 5. Non-Conventional Membrane Processes

Russo [55] studied the reclamation of three-phase OMW (Italy) by preliminary MF followed by two-stepped UF (6 kDa followed by 1 kDa membranes) and final RO operation. Best productivities upon lowest fouling were 50 L/hm^2^ for VRF equal to 3 for the ceramic MF membrane, whereas between 10–15 L/hm^2^ for UF with polymeric membranes of the MF permeate, and up to 35 L/hm^2^ when ulfrafiltering the UF permeate with 1 kDa ceramic membranes. Finally, 20–25 L/hm^2^ were yielded by the RO membrane. 

The author reported high concentration of low molecular weight (LMW) polyphenols (349 ppm from initial 55 ppm in the raw OMW samples) in the MF permeate, 76% being hydroxytyrosol. Finally, the RO retentate stream contained enriched and purified LMW polyphenols, proposed by Russo for food, pharmaceutical or cosmetic industries, whereas MF and UF retentates were suggested as fertilizers or for production of biogas in anaerobic reactors. 

Garcia-Castello *et al.* [56] proposed another treatment method for three-phase OMW in Italy, comprising MF, NF, and finally vacuum membrane distillation (VMD) or osmotic distillation (OD). MF ensured 91% and 26% reduction of suspended solids and total organic carbon (TOC), respectively, as well as 78% recovery of the initial content of polyphenols in the permeate stream. Further, NF achieved the recovery of most polyphenols in the permeate, which was enriched by ulterior treatment by osmotic distillation (OD) or vacuum membrane distillation (VMD). 

A final solution containing about 0.5 g/L free LMW polyphenols, with hydroxytyrosol representing 56% of the total amount, was produced by using calcium chloride dihydrate solution as brine. The authors highlighted the interest of these formulations for food, cosmetics, and pharmaceuticals.

Recently, Gebreyohannes *et al.* [57] investigated the treatment of three-phase OMW (Italy) by forward osmosis (FO) with a cellulose acetate membrane. They proposed a single-step FO operated with 3.7 m MgCl_2_ draw solution and 6 cm/s crossflow velocity. This treatment provided a volume reduction of 71%, complete decolorization of the permeate, and more than 98% rejection to OMW components, including biophenols and ions, thus making FO more attractive from the point of view of its cost-efficiency. Moreover, the authors studied a MBR-based pre-treatment prior to FO, which reduced pectins concentration by 92.3%, thus resulting in 30% flux enhancement.

The authors addressed the fouling issues occurring on the used membrane, and reported that 95% pure water permeability could be recovered by applied a cleaning cycle based on osmotic back-flushing, after continuous OMW dehydration tests carried out for 200 h. 

As pointed by these authors, various studies have shown that fouling occurring in FO is in most part reversible, as a result of the low foulant compaction given the negligible operating pressure gradient. Hence, FO holds a great potential to treat wastewater streams such as OMW, which has high fouling propensity. However, still low permeate productivities were reported in this research paper, and a certain permeate flux loss due to membrane fouling upon increasing the volume recovery was always noticed. Therefore, further investigation should be performed, but FO may be a promising technique for OMW reclamation.

## 6. Fouling Mitigation and Control in Membrane Treatment of OMW

In order to successfully implement a membrane process for a specific application at industrial scale, the prediction of the performance of the selected membrane is mandatory. Here an additional difficulty is given by concentration polarization and membrane fouling phenomena occurring over the membrane during the operation time, which alter the membrane output continuously.

Fouling mitigation and control is mandatory to ensure the competitiveness and economic efficiency of membrane technology. This is especially relevant in the case of membrane processes applied for the treatment and reclamation of wastewater streams like OMW, where the added value of the product, that is purified water, is low, and thus key are costs.

Fouling reduces the membrane performances in time and this leads to energy costs increments and premature substitution of the membrane modules. Since wastewater treatment must imply low operating costs, the substitution of the membrane modules cannot be performed frequently. Consistent fouling inhibition methods may assure this result, thus making membrane processes for OMW streams treatment both technically and economically feasible.

One of the possible solutions to increase the reliability of a process is the use of stable control systems. In the case of most membrane processes, this is performed by simple control strategies that do not include knowledge and control of fouling. The complete lack of advanced control systems in membrane technologies, capable of taking fouling issues into account, limits the reliability of the technology and represents one key problem to be solved to permit its further maturation. In order to achieve this result, the fouling behaviour of the system must be estimated *a priori*.

In the last years, Stoller and Ochando have successfully applied the concepts of the critical and threshold flux for fouling and process control during membrane treatment of OMW from both two-phase and three-phase systems [58,59,60,61,62,63].

The concept of the critical flux was theoretically proven and physically explained by Bacchin *et al.* [64], who proposed the first theoretical model giving explanation to membrane transport phenomena of colloidal particles. They gave a first definition of the critical flux, stating that it is the performance point above which the repulsive barrier is overcome, and below which no fouling occurs. Afterwards, Field *et al.* [65] gave an empirical approach of the concept of the critical flux for MF membranes, defining it as the permeate flux which can be successfully attained without incurring in fouling formation during the operation time. Later on, this concept was also extended to UF and NF membranes [66].

Nevertheless, some authors noted that critical flux behavior is not always observed strictly in all membrane separation processes, pointing to the idea that it might not be possible to completely inhibit fouling during the operation of some liquid-liquid membrane systems, and the case of wastewater treatments was indicated as a clear example [67,68,69]. These researchers noticed that fouling was unavoidable to a certain extent at every operating condition, and thus the concept of the threshold flux was introduced [66]. In contrast with the critical flux, the threshold flux instead makes reference to the maximum permeate flux at which fouling builds up at a very low and constant rate, and above which the rate of fouling becomes exponentially increased. In other words, the threshold flux divides a low fouling region from a high fouling region of pressure-driven membrane processes. Recently, Stoller and Ochando [69] verified the validity of this theory in the treatment of OMW by UF and NF membranes.

Long-term studies on the treatment of OMW are also relevant to examine the efficacy and cost-efficiency of membrane processes applied for the reclamation of these effluents in the long run. In this regard, Stoller published a very valuable work on a three-year long experience of effective fouling inhibition by threshold flux based optimization methods on a NF membrane module for OMW treatment [70]. The used NF membrane module could be successfully operated for three years during pilot-scale work. This could be accomplished by the adoption of appropriate fouling inhibition control, relaying on both critical flux measurements and the development of an optimized operation method. The critical flux theory was successfully applied to this system, but was not capable to explain the observed fouling behavior of the examined membrane, which could be explained instead by the threshold flux model.

Moreover, Stoller *et al.* reported in a recent paper [59] a reliable method for the conversion of critical flux measurement data into threshold flux measurements of a NF membrane module in the treatment of three-phase OMW. Stoller highlighted the important need to develop methods capable of measuring threshold fluxes quickly, and to convert critical flux measurement data into threshold flux data.

In a recent work, both critical and threshold flux concepts, which share many common aspects, were merged for simplification purpose by Stoller and Ochando into a new concept, the boundary flux, introduced in year 2014 [71]. The authors underlined that the knowledge of real-time boundary flux values is a key factor to design stable control systems for membrane processes, since operation within sub-boundary flux conditions avoids irreversible fouling and thus premature technical (and economical) failures. The boundary flux values are sensibly influenced by those parameters affecting the critical and threshold fluxes, that is: hydrodynamics, temperature, membrane properties, time, and feedstock characteristics. This concept was successfully applied in the treatment of OMW [72,73].

In particular, in the case of batch membrane treatments used to reduce the required membrane area, the fact that the bulk becomes increasingly concentrated during operation increases the difficulty in setting the adequate operating conditions. In these cases, Stoller and Ochando [71,72,73] point that the boundary flux value estimated at the end of the batch should be adopted as the target flux value initially. A deeper analysis on the concept of the boundary flux for both batch and continuous membrane operations can be found in the Boundary Flux Handbook [71].

In view of the available research papers on fouling issues in the treatment of OMW by membranes, future investigation on the fouling mechanisms occurring during membrane treatments of OMW is needed in order to fully understand the interactions among the effluent particles and the membrane surface. In this regard, the critical and threshold models, gathered in the boundary flux theory, seems a good approach which should be further studied.

## 7. Conclusions

In the present work, a review on the actual state of the art on the treatment of olive mill wastewaters (OMW) by membrane technologies is addressed. The study focuses on microfiltration (MF), ultrafiltration (UF), nanofiltration (NF), and reverse osmosis (RO), as well as membrane bioreactors (MBR) and other non-conventional membrane processes such as vacuum distillation (VD), osmotic distillation (OD), and forward osmosis (FO). 

A wide range of reclamation treatments as well as integrated processes for the treatment of OMW have already been proposed and developed by scientists and engineers, but not led to completely satisfactory results due to cost-ineffectiveness. The olive oil industry in its current status, composed of little and dispersed factories, cannot bear such high costs. Furthermore, these treatments are not able to abate the high concentration of dissolved ionic concentration present in these effluents.

Membrane processes are becoming extensively used to replace many conventional processes in the purification of water and groundwater as well as in the reclamation of wastewater streams of very diverse sources, such as those generated by agro-industrial activities. However, only a limited number of studies have been published so far regarding OMW treatment by membrane technologies. 

As it can be asserted from the present review, there are still some unresolved problems that slow down large-scale membrane applications with respect to OMW management, despite the promising perspectives. A brief insight into inhibition and control of fouling by properly-tailored pretreatment processes upstream the membrane operation and the use of the critical and threshold flux theories is also highlighted as important to ensure the cost-effectiveness of the treatment of OMW by membrane processes when transferred to the industrial scale. With respect to this, several membrane materials, configurations and pore sizes have been tested up to now, and also different pretreatments prior to membrane operations. These pretreatments provide different organic concentration abatement and also shift the particle size distribution of the colloidal and suspended matter differently. The latter is also a relevant factor: according to the pore blocking model, particles of certain sizes tend to cause fouling issues in a short time, which are those having a size similar to that of pores. 

As future tasks, research on cost-effective OMW pretreatments, either new ones or more efficient combinations of those already existing, should keep on to ensure steady state efficiencies, and further research on optimized operating conditions for each integrated pretreatment-membrane operation, as well as improvement of the existing membrane materials and new ones, which may help in enhancing the process’ economic efficiency. In this regard, the boundary flux theory seems a good approach to be further studied.
